# Tissue-resident memory CD8+T cells might enhance HBV DNA clearance in CHB patients with MASLD complication and normal ALT via the CCL-CCR pathways

**DOI:** 10.3389/fcimb.2025.1653566

**Published:** 2025-09-22

**Authors:** Chao Cai, Wen-Xuan Shang, Si-Jie Zheng, Chen Chen, Ke Xu, Lu Chen, Ya-Jie Wang, Yu-Qun Wang, Rui-Cong Chen, Yi-Jing Cai, Hui-Fang Zhang, Chen Huang, Ji Lin, Bang-Guo Zhou, Xiu-Li Lin, Lei Zhang, Ming-Qin Lu

**Affiliations:** ^1^ Department of Infectious Diseases, The First Affiliated Hospital of Wenzhou Medical University, Wenzhou, Zhejiang, China; ^2^ Department of Information Technology (IT), The First Affiliated Hospital of Wenzhou Medical University, Wenzhou, Zhejiang, China; ^3^ Department of Medical Laboratory, The First Affiliated Hospital of Wenzhou Medical University, Wenzhou, Zhejiang, China; ^4^ Department of Radiology, The First Affiliated Hospital, Zhejiang University School of Medicine, Hangzhou, Zhejiang, China; ^5^ Consortium for Infection and Innovation (CII), The First Affiliated Hospital of Wenzhou Medical University, Wenzhou, Zhejiang, China

**Keywords:** chronic hepatits B, metabolic dysfunction-associated steatotic liver disease, antiviral therapy, liver biopsy, single-cell RNA sequencing

## Abstract

**Background:**

HBV infection continues to pose a significant global health challenge, particularly in patients with normal alanine aminotransferase (ALT) level. The increasing prevalence of Metabolic dysfunction-associated steatotic liver disease (MASLD) among individuals with chronic hepatitis B (CHB) also presents new complications in management. The study aimed to investigate the relationship between MASLD and HBV DNA clearance in CHB patients with normal ALT.

**Methods:**

403 patients with ALT levels below the normal threshold who underwent liver biopsy at our institution and subsequently received antiviral therapy with NAs were retrospectively examined. Among these, 177 patients were diagnosed with MASLD. Further single cell data analysis was conducted on GSE192740 and GSE182159.

**Results:**

CHB patients concurrent with MASLD had a higher probability of achieving HBV DNA clearance. The proportion of CD8+Teff-GZMH cells was increased in the MASLD group. The interactions among CD8+ Trm-CD69 cells and other T cell subtypes were enhanced, especially within the active CCL-CCR pathways. An elevated proportion of Monocyte-THBS1 was also observed.

**Conclusions:**

CD8+ Trm-CD69 T cells may be activated by Monocyte-THBS1 cells, thereby stimulating the immune system via the CCL-CCR signaling axis. This activation facilitates the recruitment of immune cells and enhances the clearance of hepatitis B virus (HBV).

## Introduction

Hepatitis B virus (HBV) infection remains one of the most significant global health challenges, representing a major public health issue due to its substantial disease burden (GBD 2019 Hepatitis B Collaborators, 2022). In 2016, the World Health Organization (WHO) established the goal of eliminating viral hepatitis as a public health threat by 2030 (World Health Organization, 2016). In alignment with this objective, researchers worldwide have made considerable strides in the clinical diagnosis and treatment of chronic hepatitis B (CHB). However, achieving clinical cure for hepatitis B continues to present a formidable challenge, necessitating further research to overcome existing barriers.

In this context, the serological clearance of HBV DNA has emerged as a secondary target, with evidence suggesting that this clearance can significantly reduce the incidence of liver cirrhosis and hepatocellular carcinoma, thereby improving long-term patient outcomes (Liu et al., 2014). Advances in antiviral therapies have made serological of HBV DNA clearance more attainable; nonetheless, a subset of patients still experiences poor serological response, especially those with alanine aminotransferase (ALT) levels at the lower limit of normal prior to treatment.

Recent changes in dietary habits and lifestyle have led to a shifting spectrum of liver diseases, notably an increasing prevalence of metabolic dysfunction-associated steatotic liver disease (MASLD) (Riazi et al., 2022). In light of the lack of breakthroughs in curing chronic hepatitis B, the population of CHB patients coexisting with MASLD has been steadily rising (Mak et al., 2024). This unique overlap has garnered attention from researchers, with studies indicating that CHB patients with MASLD may have a higher likelihood of achieving HBV DNA clearance during antiviral therapy (Huang et al., 2024). Conversely, other research has suggested that MASLD may not serve as a favorable factor for HBV DNA clearance in CHB patients (Yang and Wei, 2022). Despite these divergent viewpoints, the molecular and biological mechanisms underlying these observations remain underexplored.

Single-cell RNA sequencing (scRNA-seq) has emerged as an innovative technique that can provide high-resolution insights into the specific cellular populations influencing disease pathophysiology. Through single-cell transcriptomic analysis, we may elucidate the distinct immune microenvironment in the livers of CHB patients with MASLD, potentially revealing novel therapeutic targets for curing CHB.

In this retrospective study, we analyzed 403 patients with ALT levels below the normal threshold who underwent liver biopsy at our institution and subsequently received antiviral therapy with nucleos(t)ide analogs (NAs), with at least one-year follow-up data. Among these, 177 patients were diagnosed with MASLD. We compared the rates of serological HBV DNA clearance after one year of antiviral treatment, revealing that CHB patients with MASLD exhibited a higher probability of achieving HBV DNA clearance. To further investigate the underlying mechanisms, we merged publicly available single-cell transcriptomic data from CHB patients with MASLD. This analysis revealed an increased proportion of CD8+ Teff-GZMH cells in MASLD patients, suggesting heightened T cell activity, along with enhanced interactions among CD8+ Tc-PDCD1 and CD8+ Trm-CD69 cells, particularly within the active CCL-CCR pathways. These findings may elucidate why coexisting MASLD serves as a favorable factor for HBV DNA clearance in CHB patients and could provide pivotal insights and potential targets for future clinical research aimed at curing CHB.

Therefore, we hypothesize that the co-existence of MASLD creates a unique intrahepatic immune microenvironment, characterized by the activation of tissue-resident memory CD8+ T cells (TRM) and enhanced chemokine signaling via CCL-CCR pathways, which promotes immune-mediated clearance of HBV DNA. This study integrates clinical outcomes from a cohort of CHB patients with normal ALT and MASLD with single-cell transcriptomic analysis to investigate this mechanistic hypothesis and identify potential immune drivers of viral clearance.

## Materials and methods

### Clinical patients and information collection

A retrospective analysis was conducted on 697 patients who underwent liver biopsy at the First Affiliated Hospital of Wenzhou Medical University between January 2017 and December 2021. The study population consisted of HBsAg-positive who had normal ALT (≤ULN, 40 U/L) for at least 6 months. All patients had been histologically diagnosed through liver biopsy to determine the inflammation grades and fibrosis stages to assist in the decision for the initiation of antiviral treatment. All enrolled patients received standardized antiviral therapy in strict accordance with the 2022 Chinese Medical Association Guidelines for Chronic Hepatitis B Management, with nucleos(t)ide analogues (NAs) administered as first-line treatment. The therapeutic regimen was uniformly implemented across all participating clinical centers, ensuring consistency in clinical practice. For the diagnosis of MASLD, the criteria proposed by the multi-society consensus in 2023 was used3. These include evidence of hepatic steatosis by liver biopsy, combined with one of the following: (a)BMI≥25kg/m2 or waist circumference>94/80 cm in men and women, respectively, (b)HbA1c≥5.7%, (c)systolic blood pressure≥130 mmHg or diastolic blood pressure≥85mmHg, (d)plasma triglycerides≥150 mg/dL and (e)plasma HDL cholesterol<40 mg/dL for men and<50 mg/dL for women. Patients with coinfection of HIV, HCV or HDV, or infection with EBV and CMV, as well as those who had received antiviral therapy or had other causes of liver damage (such as schistosomiasis liver disease, Wilson disease, drugs, autoimmune hepatitis, etc.) were excluded. A total of 403 patients met the eligibility criteria for this study. All relevant clinical data, including laboratory, histological, imaging, and other data, were retrospectively collected using a structured data frame by reviewing medical records. The assessment of liver inflammation grade and fibrosis stage for each section of the liver was performed by two experienced pathologists using Ishak depending on the scoring criteria set by the hospital at that time. The pathologists were blinded to the clinical and biochemical data of the patients. The laboratory tests conducted prior to liver biopsy during hospitalization were considered as the baseline measurements.

### Statistical analysis of clinical data

All statistical analyses were carried out using the software program SPSSv26.0 (IBM Corporation). Continuous variables were expressed as the median and inter quartile range (IQR), whereas categorical variables were shown as counts and percentages. Continuous variables were compared using independent-group Student’s t tests or the Mann–Whitney U tests and categorical variables were compared using chi-squared tests. The factors predicting HBV DNA 1-year clearance were identified by multivariate logistic regression analysis. Variables with a p value <0.05 in univariate analysis were selected into multivariate analysis by input step. A two-tailed p value of <0.05 was considered statistically significant.

### Data source for single-cell RNA sequencing

Single cell sequencing was based on the data downloaded from GSE192740 (Krenkel et al., 2020) and GSE182159 (Zhang et al., 2023) in Gene Expression Omnibus (GEO) database. Liver tissues of 4 MASLD patients and 17 HBV-infected patients were chosen. 17 patients infected with hepatitis B virus (HBV) were categorized into distinct clinical phases: immune tolerant (IT), immune active (IA), acute resolved (AR), chronic resolved (CR), and a group of normal liver (NL). The following analysis chose 3 clinical phases and adjusted the name of HC as needed: IA (Immune Activation, n=5), IT (Immune Tolerant, n=6) and NL (Normal Control, n=6). It should be noted that there are no samples classified as MASLD in the dataset GSE192740. Based on the available clinical information and in accordance with the multisociety Delphi consensus statement on the new nomenclature for fatty liver diseases, the NASH samples within GSE192740 meet the diagnostic criteria for MASLD. To maintain consistency in terminology, we refer to the samples in this dataset as MASLD throughout this manuscript. Sequencing data were imported in R software version 4.4.0 using Seurat (version 4.0) package.

### Pre-processing and quality control

Seurat object filtered out genes of ATP and ribosomal protein firstly. Then it underwent NormalizeData() and ScaleData() function. Quality control was carried out according to the criteria: nCount_RNA < 10000 and nFeature_RNA > 200 and nFeature_RNA < 2500. FindVariableFeatures() function was used to identify high variable genes with the parameters “selection.method = ‘vst’,nfeatures = 3000”. Then RunPCA() function was used to performe dimension reduction.

### Batch effect removal and analysis visualization

In order to remove batch effects discovered in PCA map, R package Harmony was performed. FindAllMarkers function was implemented to screen out differentially expressed genes for each cluster within the Seurat R package. The Umap visualization was adopted for presenting the final clustering of all cells, T cells subtypes and Macrophage subtypes using the same parameters as above. Markers of each cluster were illustrated by scCustomize R package. Heatmap of markers was made by dittoSeq R package.

### DEGs identification and cellchat analysis

FindMarkers was performed to identify highly expressed genes in subsets of T cells and macrophage between different groups. To elucidate the biological functions of these genes Gene Ontology (GO) and Kyoto Encyclopedia of Genes and Genomes (KEGG) analysis were applied to identify highly expressed genes in subsets of T cells and macrophage between different groups, two gene enrichment analysis were applied: Gene Ontology(GO) and Kyoto Encyclopedia of Genes and Genomes(KEGG) analysis. These two analysis respectively adopted enrichGO function and enrichKEGG function, which needed the same R package named clusterProfiler. Cell to cell communication analysis was carried out by CellChat R package in search of active pathways. The dot plot was portrayed by netVisual_bubble () function and the heatmap was drawn by netVisual_heatmap () function.

## Results

### Overview of clinical patient characteristics

This cohort included 403 patients with CHB, of whom 177 (44%) co-exist with MASLD (**Table 1**). Compared with the non-MASLD group, CHB patients with MASLD were significantly older, predominantly male, and had higher body mass index (BMI). In terms of viral factors, the MASLD group exhibited a lower proportion of hepatitis B e antigen (HBeAg) positivity (36.72% vs. 49.12%; P=0.013) and lower HBV DNA levels (4.90 ± 2.14 vs. 5.62 ± 2.30; P=0.002) compared to the non-MASLD group. Regarding the laboratory parameters, MASLD patients had elevated ALT and higher cholesterol levels than non-MASLD patients. Details of laboratory data are shown in **Table 1**.

**Table 1:** Baseline clinical characteristics of 403 HBV-infected patients.

	Non-MASLD	MASLD	*P*-value
Age	39.26 ± 9.05	43.52 ± 9.31	<0.001
Sex, n (%)			<0.001
Female	145 (64.16%)	69 (38.98%)	
Male	81 (35.84%)	108 (61.02%)	
BMI	21.21 ± 2.52	24.27 ± 2.64	<0.001
HBV DNA (log IU/mL)	5.62 ± 2.30	4.90 ± 2.14	0.002
PLT	211.67 ± 61.13	215.46 ± 57.58	0.527
TBL	11.94 ± 6.08	10.81 ± 4.89	0.046
AST	26.84 ± 10.61	27.94 ± 9.11	0.272
ALT	23.09 ± 8.42	27.89 ± 7.63	<0.001
GGT	21.15 ± 15.32	28.17 ± 15.19	<0.001
Cholesterol	4.88 ± 1.04	5.05 ± 1.06	0.109
Triglycerides	1.18 ± 0.87	1.73 ± 0.99	<0.001
HDL	1.40 ± 0.33	1.16 ± 0.28	<0.001
HBeAg, n (%)			0.013
negative	115 (50.88%)	112 (63.28%)	
positive	111 (49.12%)	65 (36.72%)	

Data are presented as a mean value with SD.

ALT, alanine aminotransferase; AST, aspartate aminotransferase; BMI, body mass index; GGT, gamma-glutamyl transferase; HBV DNA, Hepatitis B virus-deoxyribonucleic acid; HBeAg, hepatitis B e antigen; HDL, high-density lipoprotein; MASLD, metabolic dysfunction-associated steatotic liver disease; PLT, platelet; TBL, total bilirubin.

### Pathological feature and prognosis comparison

The proportion of patients with significant histological disease (significant liver necroinflammation and/or fibrosis) was significantly higher in the MASLD group compared to the non-MASLD group (68.36% vs 58.85%, p = 0.050, **Table 2**). Similarly, the proportions of significant liver necroinflammation and fibrosis also showed this trend (63.84% vs 51.77%, p=0.015; 38.98% vs 27.43%, p=0.014, **Table 2**). As a retrospective study, the exact scoring system adopted for each patient depended on the criteria (Ishak) selected by the hospital at that moment.

**Table 2:** Significance comparison of pathological change in liver and serum HBV DNA clearance rate between Non-MASLD and MASLD group.

	Non-MASLD 226	MASLD 177	*P*-value
	Liver Pathological Feature		
Necroinfammation, n (%)			0.015
non-significant	109 (48.23%)	64 (36.16%)	
significant	117 (51.77%)	113 (63.84%)	
Fibrosis, n (%)			
non-significant	164 (72.57%)	108 (61.02%)	0.014
significant	62 (27.43%)	69 (38.98%)	
Damage^†^, n (%)			0.050
non-significant	93 (43.15%)	56 (31.64%)	
significant	133 (58.85%)	121 (68.36%)	
	Serum HBV DNA Clearance		
HBV DNA negative, n (%)			0.002
Yes	135 (59.73%)	132 (74.58%)	
No	91 (40.27%)	45 (25.42%)	

MASLD, metabolic dysfunction-associated steatotic liver disease.

^**†**^Liver damage (Necroinfammation and/or Fibrosis).

All patients received antiviral treatment and followed up for at least one year. There were 132 (74.58%) and 135 (59.73%) patients who achieved HBV DNA clearance in the MASLD and non-MASLD groups, respectively (**Table 2**).

### Independent predictors for HBV clearance

We performed univariate logistic regression analysis in patients who completed regular follow-up and identified age, BMI, MASLD, gamma-glutamyl transferase (GGT), and liver damage as predictors of antiviral treatment response (**Table 3**) In contrast, HBV DNA, HBeAg, platelet count (PLT), and cholesterol levels were inversely associated with treatment response. Further multivariate logistic regression analysis, HBV DNA (OR=0.524, 95%CI, 0.410-0.669), HBeAg (OR=0.184, 95%CI, 0.069-0.492), Cholesterol (OR=0.665, 95%CI, 0.480-0.922) and BMI (OR=0.855, 95%CI, 0.746-0.981) were inversely associated with HBV DNA clearance. Conversely, age (OR=1.043, 95%CI, 1.003-1.085), MASLD (OR=2.209, 95%CI, 1.018-4.797) and liver damage (OR=3.314, 95%CI, 1.496-6.567) were independent predictors of antiviral treatment response. Notably, after adjusting for confounders, MASLD was associated with an increased rate of treatment response (OR = 2.209, 95% CI: 1.018–4.797).

**Table 3:** Univariate and multivariable logistic regression analyses for identifying predictors of significant HBV DNA clearance.

	Univariate			Multivariate		
	OR	95% CI	P-value	OR	95% CI	P-value
Age	1.069	(1.043,1.096)	<0.0001	1.043	(1.003,1.085)	0.03638
Male	1.418	(0.934,2.152)	0.10102			
BMI	1.104	(1.027,1.187)	0.00701	0.855	(0.746,0.981)	0.02567
MASLD	1.977	(1.286,3.040)	0.00190	2.209	(1.018,4.797)	0.04505
HBV DNA	0.400	(0.340,0.470)	<0.0001	0.524	(0.410,0.669)	<0.0001
HBeAg positive	0.029	(0.016,0.055)	<0.0001	0.184	(0.069,0.492)	0.00074
PLT	0.995	(0.991,0.998)	0.00304	0.999	(0.991,0.998)	0.00304
TBL	1.001	(0.965,1.039)	0.96389			
ALT	1.021	(0.996,1.046)	0.10407			
GGT	1.020	(1.003,1.036)	0.01727	1.005	(0.984,1.027)	0.62060
Triglycerides	1.057	(0.842,1.328)	0.63137			
HDL	0.615	(0.323,1.170)	0.13864			
Cholesterol	0.749	(0.609,0.920)	0.00595	0.665	(0.480,0.922)	0.01424
Damage	2.191	(1.432,3.351)	0.00030	3.134	(1.496,6.567)	0.00248

ALT, alanine aminotransferase; AST, aspartate aminotransferase; BMI, body mass index; GGT, gamma-glutamyl transferase; HBV DNA, Hepatitis B virus-deoxyribonucleic acid; HBeAg, hepatitis B e antigen; HDL, high-density lipoprotein; MASLD, metabolic dysfunction-associated steatotic liver disease; PLT, platelet; TBL, total bilirubin.

Previous studies and our logistic regression analysis identified several factors (Log10DNA, Age, Sex, BMI, HBeAg, GGT, Cholesterol, PLT, TBIL, AST, ALT, Damage, Triglycerides, and HDL) potentially associated with HBV DNA clearance. To assess whether MASLD modifies the effects of these variables, we performed interaction tests. As shown in **Table 4**, no significant interactions were observed between MASLD and these factors (p-values for all interactions > 0.05), indicating that the association between MASLD and HBV DNA clearance is independent of these covariates.

**Table 4:** Statistical interactions between MASLD and baseline characteristics.

		Multivariate			
		OR^‡^	95%CI	*P-value*	*P*_interaction_
Age	Lowest tertile	2.20	(0.42,11.56)	0.3506	0.7466
	Middle tertile	6.37	(0.72,56.62)	0.0965	
	Highest tertile	2.09	(0.42,10.46)	0.3706	
Sex	Female	3.30	(0.84,12.93)	0.0868	0.4323
	Male	1.60	(0.49,5.26)	0.4399	
BMI	<23	1.69	(0.42,6.81)	0.4625	0.3767
	≥23	4.00	(1.07,14.89)	0.0390	
HBVDNA	Lowest tertile	10.46	(0.59,183.97)	0.1085	0.4130
	Middle tertile	1.52	(0.29,7.87)	0.6180	
	Highest tertile	5.02	(1.03,24.39)	0.0456	
HBeAg	Negative	3.62	(0.68,19.22)	0.1304	0.8766
	Positive	4.28	(1.20,15.26)	0.0250	
Cholesterol	<5.2	2.83	(0.85,9.50)	0.0913	0.8349
	≥5.2	2.31	(0.51,10.50)	0.2799	
Damage	No	4.24	(0.97,18.44)	0.0543	0.3597
	Yes	1.83	(0.57,5.85)	0.3082	

^‡^ORs were adjusted for age, sex, BMI, HBeAg positive, HBV DNA, GGT; Cholesterol, PLT, TBL, AST, ALT, Liver Damage, Triglycerides, and HDL.

ALT, alanine aminotransferase; AST, aspartate aminotransferase; BMI, body mass index; GGT, gamma-glutamyl transferase; HBV DNA, Hepatitis B virus-deoxyribonucleic acid; HBeAg, hepatitis B e antigen; HDL, high-density lipoprotein; MASLD, metabolic dysfunction-associated steatotic liver disease; PLT, platelet; TBL, total bilirubin.

### Data acquirement and preliminary single-cell sequencing analysis

**Figure 1:**
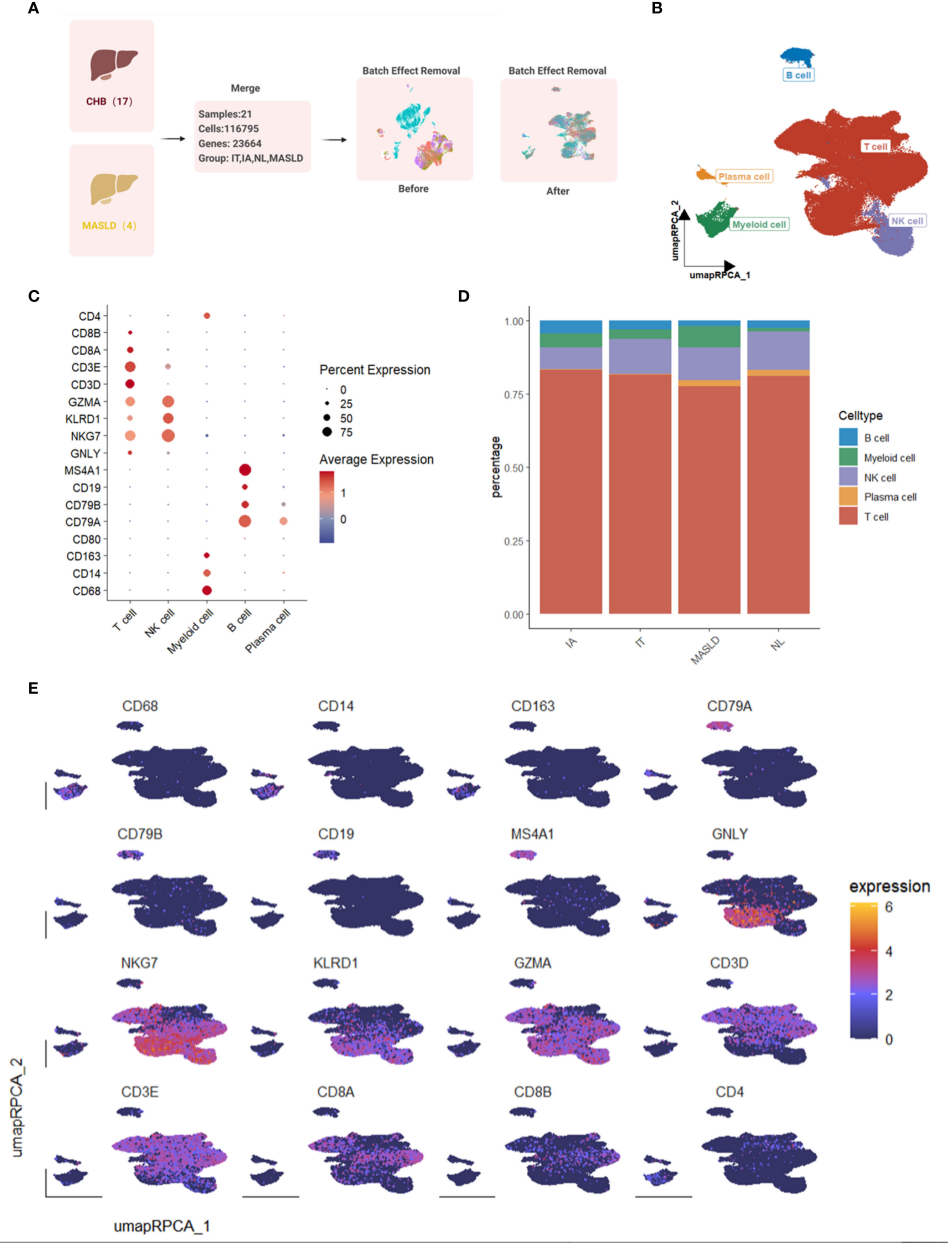
Single-cell transcriptome map of immune cells in livers from 17 HBV-infected patients and 4 patients with MAFLD. **(A)** Workflow of single-cell sequencing datasets merging. **(B)** UMAP plot of 116,795 immune cells from hepatic tissue samples with HBV or MAFLD. **(C)** Dot plot of marker gene expression levels in each group. **(D)** Bar plot of cell proportions in each group. **(E)** Feature plots of marker expressions.

We conducted single-cell sequencing analysis using data downloaded from GEO datasets. A total of 116,795 cells from 21 liver tissue samples, comprising 17 samples infected with HBV and 4 samples with MASLD, were included in the study (**Figure 1A**). Samples from HBV-infected patients were categorized into three groups: immune active (IA, n=5), immune tolerant (IT, n=6), and normal control (NL, n=6). Data from all 21 samples were combined to create a general Seurat object. Following preprocessing and dimension reduction, cells were clustered and identified as five cell types, including T cells, NK cells, B cells, myeloid cells, and plasma cells (**Figure 1B**). The expression levels of marker genes were displayed in dot plots and feature plots (**Figures 1C, E**). The proportion of each cell type in the groups was shown in bar plots. Notably, the MASLD group exhibited the highest proportion of myeloid cells (**Figure 1D**).

### T cell subtype identification

**Figure 2:**
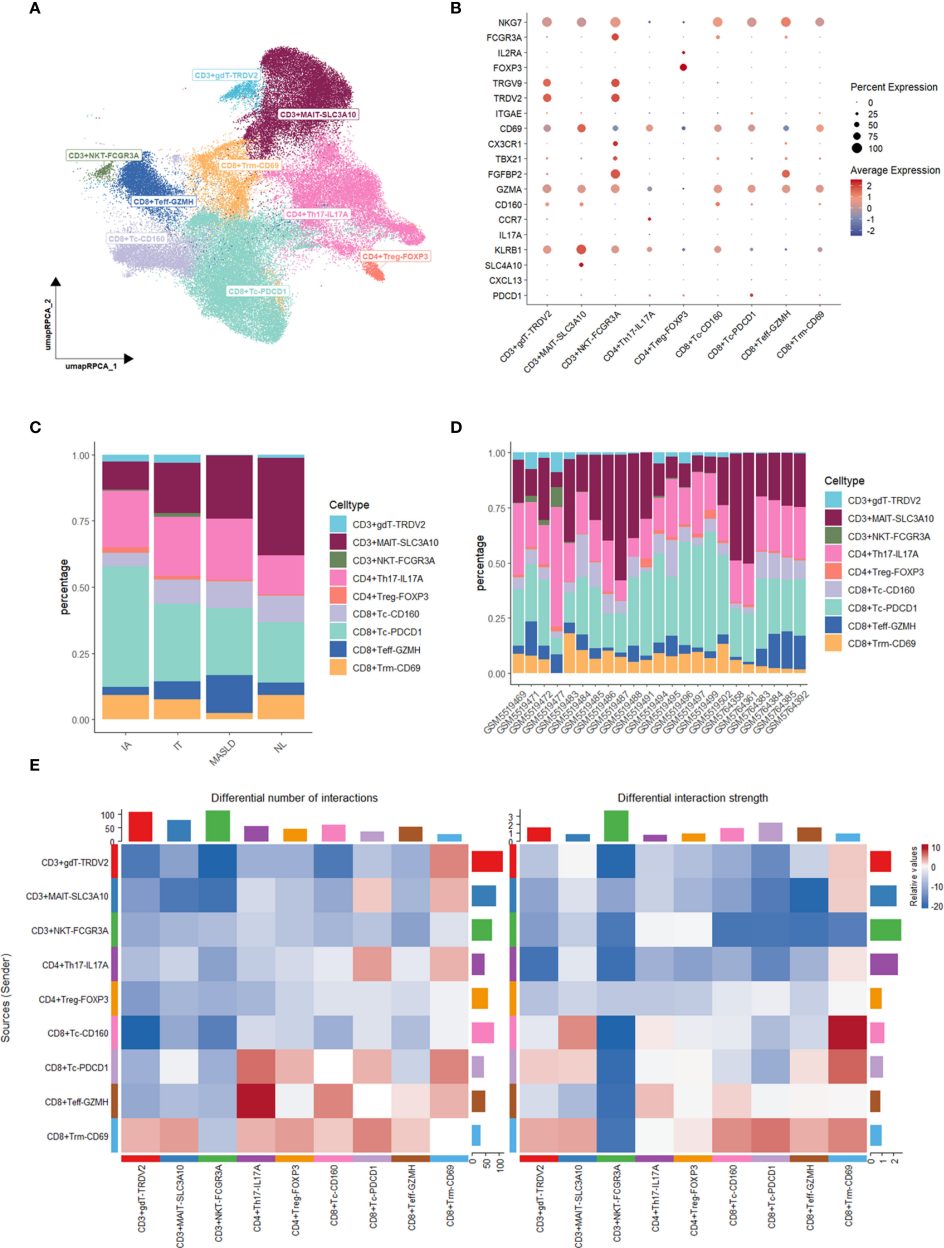
Characterization and Cellchat analysis of T cell subsets. **(A)** UMAP plot of T cell subsets. **(B)** Dot plot of marker gene expression levels in each group. **(C)** Bar plot of cell proportions in each group. **(D)** Bar plot of cell proportions in each sample. **(E)** Heatmap of differential interaction numbers and strengths.

T cell subset was further identified into 9 subtypes, including CD3+gdT-TRDV2, CD3+MAIT-SLC3A10, CD3+NKT-FCGR3A, CD4+Th17-IL17A, CD4+Treg-FOXP3, CD8T+Tc-CD160, CD8+Tc-PDCD1, CD8+Teff-GZMH, CD8+Trm-CD69 (**Figures 2A, B**). A higher proportion of CD8+ Teff-GZMH was observed in the MASLD group compared to other groups, indicating a more active inflammatory response in the MASLD group (**Figures 2C, D**).

### CCL-CCR pathways in CD8+ Trm_CD69 cells active in MASLD

**Figure 3:**
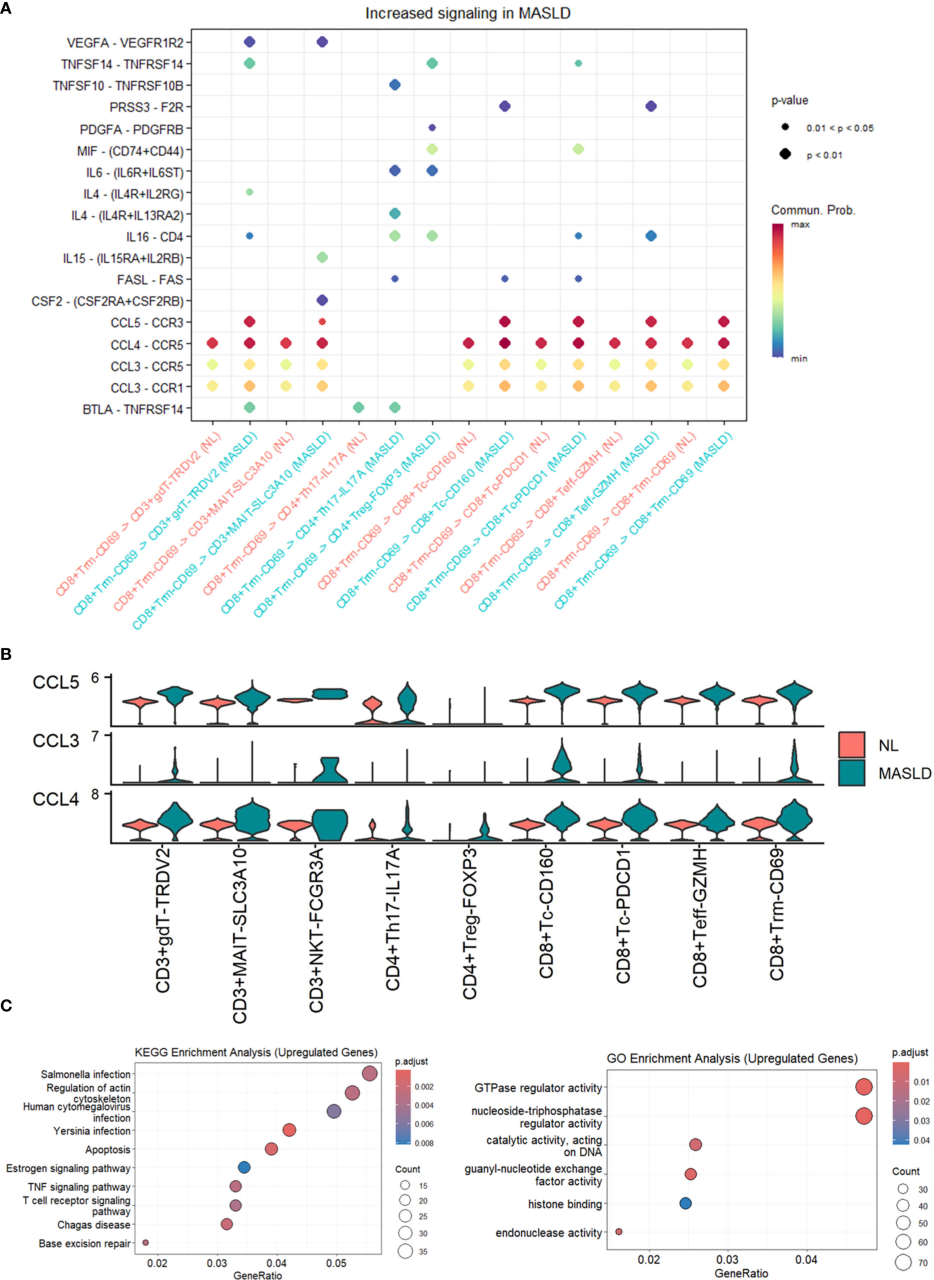
Cellchat analysis and enrichment analysis of T cell subsets. **(A)** Dot plot of increased signaling in MASLD. **(B)** Expression levels of CCL3, CCL4, and CCL5 in T cell subsets. **(C)** Dot plot of KEGG enrichment analysis and GO enrichment analysis of upregulated genes in MASLD. **(D)** Dot plot of GO enrichment analysis of upregulated genes in MASLD.

To discover how T cells made an influence, we performed Cellchat analysis among T cell subtypes. Compared MASLD group to NL group, The interactive number and strength of both CD8+ Tc-PDCD1 and CD8+ Trm-CD69 across the majority of T cell subtypes are enhanced in the context of both upstream and downstream signaling pathways (**Figure 2E**). Given the increased interaction levels of CD8+ Trm-CD69 cells with the majority of other cell types, we further analyzed the communication between CD8+ Trm-CD69 cells and other T cell subsets, detailing the specific signaling pathways involved (**Figure 3A**). Among these pathways several CCL-CCR pathways were observed highly active in MASLD group. Further gene expression analyze show CCL3, CCL4 and CCL5 gene were significantly elevated in MASLD group (**Figure 3B**).

### T cells within MASLD might benefit HBV DNA clearance

We further worked out differentially expressed genes (DEGs) between MASLD group and NL group in T cells. Under the guidance of Gene Ontology (GO) and Kyoto Encyclopedia of Genes and Genomes (KEGG), we used top genes to seek biological process and pathways. GO analysis revealed that the top DEGs in MASLD of T cells served a function in TNF signaling pathway and T cell receptor signaling pathway, both known to be closely associated with inflammation (**Figure 3C**). The outcome supported the idea that MASLD promoted HBV clearance by immune activation of T cells and laid the foundation for exploring the influencing factor in MASLD.

### Potential association between myeloid cells and HBV DNA clearance

**Figure 4:**
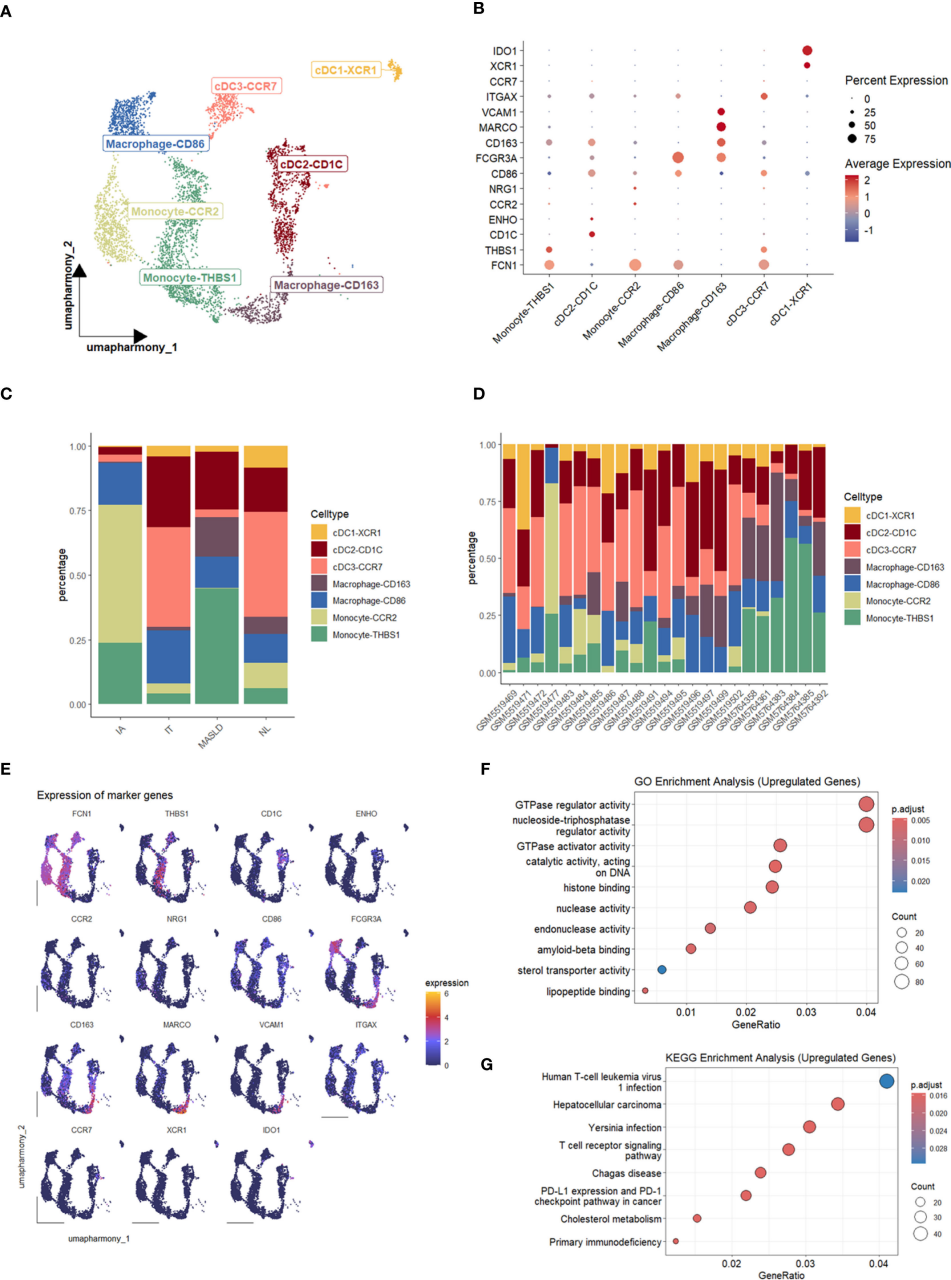
Characterization and enrichment analysis of myeloid cell subsets. **(A)** UMAP plot of myeloid cell subsets. **(B)** Dot plot of marker gene expression levels in each group. **(C)** Bar plot of cell proportions in each group. **(D)** Bar plot of cell proportions in each sample. **(E)** Feature plots of marker expressions. **(F)** Dot plot of GO enrichment analysis of upregulated genes in MASLD. **(G)** Dot plot of KEGG enrichment analysis of upregulated genes in MASLD.

Analysis of cell populations revealed a significantly elevated proportion of cells from MASLD patients (**Figure 1D**), suggesting disease-associated alterations in these cells may influence T cell activation. Thus, we paid attention to myeloid cells and identified them into 7 subtypes (**Figures 4A, B, E**), including cDC1-XCR1, cDC2-CD1C, cDC3-CCR7, Macrophage-CD163, Macrophage-CD86, Monocyte-CCR2 Monocyte-THBS1. According to Enrichment analysis, up-regulated differential genes were enriched in T cell receptor signaling pathway (**Figures 4F, G**). Our previous study identified a significant cell interaction level increasing in CD8+ Trm-CD69 cells. Combined with the result of upregulated genes enriched in the T cell receptor signaling pathway, this suggests the presence of a specific mechanism within the myeloid cells and T cells that may influence viral clearance in MASLD patients concurrent with HBV.

## Discussion

CHB remains a significant global public health challenge, and the WHO’s goal of eliminating viral hepatitis as a public health threat by 2030 places increasing demands on current treatment strategies (World Health Organization, 2016). Meanwhile, the rising prevalence of MASLD has resulted in a growing number of patients with concurrent CHB and MASLD (Mak et al., 2024). This co-occurrence presents a novel scenario for antiviral treatment in CHB patients, raising substantial interest in its potential impact on HBV DNA clearance. However, current research findings on this topic presents conflicting results.

In our study, we retrospectively analyzed a cohort of 403 CHB patients with ALT levels below the normal threshold who underwent liver biopsy at our institution and subsequently received antiviral therapy with NAs, with at least one-year of follow-up. Among these patients, 177 were diagnosed with MASLD. Baseline clinical characteristics revealed that CHB patients with MASLD exhibited significantly higher BMI (24.27 ± 2.64 vs. 21.21 ± 2.52), ALT (27.89 ± 7.63 vs. 23.09 ± 8.42), GGT (28.17 ± 15.19 vs. 21.15 ± 15.32), and Triglycerides (1.73 ± 0.99 vs. 1.18 ± 0.87), along with lower levels of HDL (1.16 ± 0.28 vs. 1.40 ± 0.33). Elevated BMI and triglyceride levels, combined with reduced HDL, suggest a higher likelihood of metabolic dysfunction in patients with MASLD. Additionally, higher baseline ALT and GGT levels indicate that CHB patients with MASLD may experience greater hepatic inflammation. Histological and biochemical analysis corroborated these findings, revealing that patients with both CHB and MASLD had higher rates of significant liver inflammation (63.84% vs. 51.77%, P=0.015), significant fibrosis (38.98% vs. 27.43%, P=0.014), and significant histological damage (68.36% vs. 58.85%, P=0.05). Previous studies have established that appropriate levels of intrahepatic inflammation can enhance the immune system’s capacity to clear infected cells, thereby improving the efficacy of antiviral therapies (Fanning et al., 2019). Notably, the results of one-year follow-up demonstrated that CHB patients with MASLD achieved a higher HBV DNA clearance rate (74.58% vs. 58.85%, P=0.002), even when their ALT levels were at the lower limit of normal. Logistic regression analysis further confirmed that with the presence of MASLD was an independent predictor of HBV DNA clearance at one year (OR: 1.977, P=0.00190). This finding aligns with the recent WHO guidelines for the prevention, diagnosis, care, and treatment of individuals with chronic hepatitis B infection, which recommend antiviral therapy in the presence of MASLD, regardless of ALT, HBV DNA, or APRI scores (World Health Organization, 2024). Nevertheless, the mechanisms underlying the enhanced antiviral treatment response in CHB patients with MASLD remain insufficiently understood. Some studies have suggested that MASLD may inhibit HBV DNA replication via the TLR4/MyD88/IFNβ signaling pathway in animal models of CHB (Zhang et al., 2015). Other research proposes that metabolic stress may attenuate the progression of chronic hepatitis B (Tourkochristou et al., 2022). However, these studies primarily focused on bulk tissue molecular biology. Given the complexity of the intrahepatic immune microenvironment in CHB patients with MASLD, there is a notable gap in research exploring immune characteristics at the single-cell level. Given the limited understanding of the intrahepatic immune microenvironment in CHB patients with MASLD and the unclear mechanisms underlying HBV DNA clearance in this population, we analyzed data from GSE192740 (Krenkel et al., 2020), a single-cell transcriptomic dataset of CHB patients, and GSE182159 (Zhang et al., 2023), a dataset of MASLD patients. After merging and batch effect correction, we identified a total of 116,795 cells categorized into five major cell types. Notably, the proportion of myeloid cells was higher in the MASLD samples compared to other groups. These myeloid cells, which include liver-resident Kupffer cells and monocytes recruited from the bloodstream, play a critical role in the regulation of various liver diseases (Khan et al., 2024). The increased proportion of myeloid cells in the MASLD group may reflect heightened intrahepatic inflammation relative to the CHB cohort without MASLD. T cells, as the predominant effector cells, constituted the largest cell population in our single-cell data across all groups. We identified nine subtypes of T cells, and interestingly, the MASLD group exhibited a marked increase in CD8+ Teff-GZMH cells and a decrease in CD8+ TRM-CD69 cells. Further Cellchat analysis of cell interaction differences between the MASLD and non-MASLD groups revealed that although the CD8+ TRM-CD69 cells constituted the smallest population in the MASLD group, their intercellular interactions were remarkably active, both in terms of number and strength. TRM cells are a recently characterized subset of memory T cells that reside at infection sites, maintaining a highly activated state and are implicated in the pathogenesis of various diseases, including infections, cancers, and autoimmune diseases (Christo et al., 2024). Virus-specific liver tissue-resident memory T (TRM) cells play a critical role in controlling viral replication by directly targeting infected hepatocytes and orchestrating local immune responses within the liver microenvironment. These cells contribute to the functional cure for HBV patients by maintaining a sustained antiviral state, even in the absence of continuous antiviral therapy. Liver TRM cells, characterized by their unique ability to persist in the liver parenchyma without recirculating, provide long-term viral control through their rapid reactivation upon antigen re-exposure and their capacity to secrete antiviral cytokines such as interferon-gamma (IFN-γ) and tumor necrosis factor-alpha (TNF-α). This localized immune surveillance mechanism is essential for preventing viral rebound and achieving durable immune-mediated control of HBV infection (Dusséaux et al., 2017; Pallett et al., 2017; Lim et al., 2019).

Furthermore, our analysis indicated that the CD8+ TRM-CD69 cells in the MASLD group engaged in heightened interactions with other cell types, particularly with CD3+NKT-FCGR3A cells. Notably, we identified significant signaling changes in the interactions between CD8+ TRM-CD69 and various T cell subtypes, with the CCL3-CCR1, CCL3-CCR5, CCL4-CCR5, and CCL5-CCR3 pathways showing the most pronounced increases. The expression levels of CCL3, CCL4, and CCL5 were also significantly elevated in MASLD compared to the non-MASLD group. Chemokines are a subclass of cytokines responsible for the transport of immune cells and the development of lymphoid tissues (Chen et al., 2018). Based on their primary protein structure and the positions of the first two cysteine residues, chemokines can be categorized into four main classes: C, CC, CXC, and CX3C, each participating in a variety of immune regulatory functions (Huang et al., 2008; Fox et al., 2015). Specifically, CCL3 and CCL4, also known as MIP-1α and MIP-1β (macrophage inflammatory proteins), are critical in attracting various immune cell types, including monocytes, T cells, and natural killer (NK) cells, to sites of inflammation and injury. CCL5, known as RANTES (Regulated upon Activation, Normal T cell Expressed and Secreted), is another multifunctional chemokine that can recruit several types of immune cells, including T cells, monocytes, eosinophils, and basophils. The heightened presence of CCL3, CCL4, and CCL5 correlates with increased immune cell recruitment and activation within the liver microenvironment, suggesting a robust inflammatory response conducive to viral elimination.

It is noteworthy that in this study, the percentage of TRM cells in the livers of patients with MASLD was lower than that in other groups. However, this lower frequency does not imply that these TRM cells are “quiescent.” On the contrary, Cellchat analysis strongly suggests that they remain highly active. This phenomenon may be attributed to the MASLD liver microenvironment—characterized by lipotoxicity, insulin resistance, endoplasmic reticulum stress, and gut dysbiosis-derived metabolites—which serves as a potent chronic low-grade inflammatory stimulus (Wang et al., 2025). Such an environment efficiently activates TRM cells and induces them to produce soluble mediators. Meanwhile, the inflammatory microenvironment in MASLD may also involve robust negative feedback mechanisms that restrain excessive TRM expansion without necessarily suppressing their potent short-term effector functions. Consequently, although their proportion is relatively low, their functional activity remains high.

In contrast, in the hepatitis B group persistent viral antigens provide a powerful signal driving the expansion and long-term maintenance of antigen-specific TRM cells, resulting in a higher TRM frequency (Liu et al., 2023). However, while HBV involves continuous high-level exposure to viral antigens (PAMPs), MASLD is driven by metabolically stressed damage-associated molecular patterns (DAMPs). The persistent high-load PAMP stimulation in HBV may lead to TRM exhaustion and functional impairment, whereas the non-specific inflammatory stimulation in MASLD can activate a broader repertoire of TRM clones, potentially endowing them with enhanced functional activity.

In our differential gene enrichment analysis of T cells, we also observed significant enrichment in pathways such as the TNF signaling pathway and the T cell receptor signaling pathway, both of which are closely linked to tissue inflammation and T cell recruitment. The active inflammation observed in MASLD tissue may be associated with an increased rate of HBV DNA clearance after one year. A sufficiently vigorous immune response could enhance virus elimination. These findings suggest that the resident CD8+ TRM-CD69 T cells in the liver tissue of patients with CHB and MASLD may activate the immune system via CCL-CCR pathways, thereby promoting immune cell recruitment and enhancing HBV clearance.

Consistent with our findings, we observed a notable presence of Monocyte-THBS1 cells within myeloid cell subpopulations in the MASLD cohort. Monocytes are a type of immune cell that can respond to invading pathogens or tissue damage in the bloodstream by migrating to the site of injury. They play a critical role in regulating the inflammatory response, presenting antigens, and interacting with other immune cells such as the CD8+ TRM-CD69 cells identified in our earlier research, thereby influencing the progression of diseases. In patients with chronic hepatitis B (CHB) complicated by MASLD, the heightened activity of CD8+ TRM-CD69 cells, coupled with an increased abundance of Monocyte-THBS1 cells, likely facilitates a synergistic effect that enhances immune responses and promotes the clearance of HBV. Furthermore, our analysis of differentially expressed genes in myeloid cells revealed significant enrichment in the T cell receptor signaling pathway. These observations lend robust support to our hypothesis that tissue-resident memory CD8+ T cells may play a crucial role in enhancing HBV DNA clearance in CHB patients with MASLD complications and normal ALT levels, potentially mediated through the CCL-CCR pathways activated by Monocyte-THBS1 cells.

However, there were several limitations to our study. First, the clinical cohort was based on a single-center sample, which may introduce bias. Additionally, the lack of single-cell transcriptomic data specifically from liver tissues of CHB patients with MASLD limits our ability to fully capture the immune characteristics of this population. Future validation through cellular, animal, and clinical studies is warranted to corroborate our findings and explore their implications, which will be a focal point in our upcoming research endeavors.

## Data Availability

Publicly available datasets were analyzed in this study. This data can be found here: Data source link: part1:https://www.jianguoyun.com/p/DUbkzeEQ7pmWDRj-yuYFIAA part2:https://www.jianguoyun.com/p/DbAplggQ7pmWDRiAy-YFIAA.

## References

[B1] Chen K. Bao Z. Tang P. Gong W. Yoshimura T. Wang J. M. (2018). Chemokines in homeostasis and diseases. Cell Mol. Immunol. 15, 324–334. doi: 10.1038/cmi.2017.134, PMID: 29375126 PMC6052829

[B2] Christo S. N. Park S. L. Mueller S. N. Mackay L. K. (2024). The multifaceted role of tissue-resident memory T cells. Annu. Rev. Immunol. 42, 317–345. doi: 10.1146/annurev-immunol-101320-020220, PMID: 38941605

[B3] Dusséaux M. Masse-Ranson G. Darche S. Ahodantin J. Li Y. Fiquet O.. (2017). Viral load affects the immune response to HBV in mice with humanized immune system and liver. Gastroenterology 153, 1647–1661.e9. doi: 10.1053/j.gastro.2017.08.034, PMID: 28851562 PMC5733397

[B4] Fanning G. C. Zoulim F. Hou J. Bertoletti A. (2019). Therapeutic strategies for hepatitis B virus infection: towards a cure. Nat. Rev. Drug Discov. 18, 827–844. doi: 10.1038/s41573-019-0037-0, PMID: 31455905

[B5] Fox J. C. Nakayama T. Tyler R. C. Sander T. L. Yoshie O. Volkman B. F. (2015). Structural and agonist properties of XCL2, the other member of the C-chemokine subfamily. Cytokine 71, 302–311. doi: 10.1016/j.cyto.2014.11.010, PMID: 25497737 PMC4297508

[B6] GBD 2019 Hepatitis B Collaborators (2022). Global, regional, and national burden of hepatitis B, 1990-2019: a systematic analysis for the Global Burden of Disease Study 2019. Lancet Gastroenterol. Hepatol. 7, 796–829. doi: 10.1016/S2468-1253(22)00124-8, PMID: 35738290 PMC9349325

[B7] Huang J. Chen K. Gong W. Dunlop N. M. Wang J. M. (2008). G-protein coupled chemoattractant receptors and cancer. Front. Biosci. 13, 3352–3363. doi: 10.2741/2930, PMID: 18508437 PMC7422331

[B8] Huang S. C. Su T. H. Tseng T. C. Chen C. L. Hsu S. J. Liu C. H.. (2024). Metabolic dysfunction-associated steatotic liver disease facilitates hepatitis B surface antigen seroclearance and seroconversion. Clin. Gastroenterol. Hepatol. 22 (3), 581–590.e6. doi: 10.1016/j.cgh.2023.09.040, PMID: 37871842

[B9] Khan M. N. Mao B. Hu J. Shi M. Wang S. Rehman A. U.. (2024). Tumor-associated macrophages and CD8+ T cells: dual players in the pathogenesis of HBV-related HCC. Front. Immunol. 15, 1472430. doi: 10.3389/fimmu.2024.1472430, PMID: 39450177 PMC11499146

[B10] Krenkel O. Hundertmark J. Abdallah A. T. Kohlhepp M. Puengel T. Roth T.. (2020). Myeloid cells in liver and bone marrow acquire a functionally distinct inflammatory phenotype during obesity-related steatohepatitis. Gut 69 (3), 551–563. doi: 10.1136/gutjnl-2019-318382, PMID: 31076404

[B11] Lim C. J. Lee Y. H. Pan L. Lai L. Chua C. Wasser M.. (2019). Multidimensional analyses reveal distinct immune microenvironment in hepatitis B virus-related hepatocellular carcinoma. Gut 68 (5), 916–927. doi: 10.1136/gutjnl-2018-316510, PMID: 29970455

[B12] Liu J. Yang H. I. Lee M. H. Lu S. N. Jen C. L. Batrla-Utermann R.. (2014). Spontaneous seroclearance of hepatitis B seromarkers and subsequent risk of hepatocellular carcinoma. Gut 63 (10), 1648–1657. doi: 10.1136/gutjnl-2013-305785, PMID: 24225939

[B13] Liu L. Liu J. Li P. Luo J. Qin R. Peng Q.. (2023). Single-cell analysis reveals HBV-specific PD-1+CD8+ TRM cells in tumor borders are associated with HBV-related hepatic damage and fibrosis in HCC patients. J. Exp. Clin. Cancer Res. 42 (1), 152. doi: 10.1186/s13046-023-02710-4, PMID: 37353792 PMC10288678

[B14] Mak L. Y. Liu K. Chirapongsathorn S. Yew K. C. Tamaki N. Rajaram R. B.. (2024). Liver diseases and hepatocellular carcinoma in the Asia-Pacific region: burden, trends, challenges and future directions. Nat. Rev. Gastroenterol. Hepatol. 21 (12), 834–851. doi: 10.1038/s41575-024-00967-4, PMID: 39147893

[B15] Pallett L. J. Davies J. Colbeck E. J. Robertson F. Hansi N. Easom N. J. W.. (2017). IL-2high tissue-resident T cells in the human liver: Sentinels for hepatotropic infection. J. Exp. Med. 214 (6), 1567–1580. doi: 10.1084/jem.20162115, PMID: 28526759 PMC5461007

[B16] Riazi K. Azhari H. Charette J. H. Underwood F. E. King J. A. Afshar E. E.. (2022). The prevalence and incidence of NAFLD worldwide: a systematic review and meta-analysis. Lancet Gastroenterol. Hepatol. 7 (9), 851–861. doi: 10.1016/S2468-1253(22)00165-0, PMID: 35798021

[B17] Tourkochristou E. Assimakopoulos S. F. Thomopoulos K. Marangos M. Triantos C. (2022). NAFLD and HBV interplay - related mechanisms underlying liver disease progression. Front. Immunol. 13, 965548. doi: 10.3389/fimmu.2022.965548, PMID: 36544761 PMC9760931

[B18] Wang X. Qiu Z. Zhong Z. Liang S. (2025). TREM2-expressing macrophages in liver diseases. Trends Endocrinol. Metab., S1043–2760(25)00084-0. doi: 10.1016/j.tem.2025.04.009, PMID: 40368708 PMC12353850

[B19] World Health Organization (2016). Global health sector strategy on viral hepatitis 2016–2021. Available online at: https://www.afro.who.int/publications/global-health-sector-strategy-viral-hepatitis-2016-2021.

[B20] World Health Organization (2024). Guidelines for the prevention, diagnosis, care and treatment for people with chronic hepatitis B infection[R] (Geneva: World Health Organization). 40424433

[B21] Yang M. Wei L. (2022). Impact of NAFLD on the outcome of patients with chronic hepatitis B in Asia. Liver Int. 42, 1981–1990. doi: 10.1111/liv.15252, PMID: 35373500

[B22] Zhang C. Li J. Cheng Y. Meng F. Song J. W. Fan X.. (2023). Single-cell RNA sequencing reveals intrahepatic and peripheral immune characteristics related to disease phases in HBV-infected patients. Gut 72 (1), 153–167. doi: 10.1136/gutjnl-2021-325915, PMID: 35361683 PMC9763233

[B23] Zhang R. N. Pan Q. Zhang Z. Cao H. X. Shen F. Fan J. G. (2015). Saturated Fatty Acid inhibits viral replication in chronic hepatitis B virus infection with nonalcoholic Fatty liver disease by toll-like receptor 4-mediated innate immune response. Hepat Mon 15, e27909. doi: 10.5812/hepatmon.15(5)2015.27909, PMID: 26045709 PMC4451278

